# 

**Published:** 1840-01

**Authors:** 


### CONTENTS OF No. XVII.

OF THE

### British and Foreign Medical Review.

JANUARY, 1840.

---

### PART FIRST.—Analytical and Critical Reviews.

PAGE

ART. I.—1. Über Entwickelungsgeschichte der Thiere. Beobachtungen und Reflexionen. Von Dr. K. E. v. BAER. Erster und Zweiter Thielen . 1
Observations and Reflections on the History of the Development of Animals. By CHARLES ERNEST VON BAER. First and Second Parts.
2. Handbuch der Entwickelungsgeschichte des Menschen: mit Vergleichender Rücksicht der Entwickelung der Säugthiere und Vögel. Von Dr. G. VALENTIN ib.
Manual of the History of the Development of Man: with a Comparative View of the Development of Mammalia and Birds By Dr. G. VALENTIN.
3. Prodromus Historiæ Generationis Hominis atque Animalium: sistens Icones ad illustrandum Ovi Primitivi, imprimis Vesiculæ Germinativæ et Germinis in Ovario inclusi, genesin atque structuram per omnes animalium Classes multos que Ordines indagatam. Auctore RUDOLPHO WAGNER, &c. &c. . . ib.
A Precursor of a History of the Generation of Man and Animals: consisting of Drawings illustrative of the Origin and Structure of the Primitive Ovum in the Ovary, especially of the germinative Vesicle and Germ; in all the Classes and many of the Orders. By RUDOLPH WAGNER, &c. &c.
4. On the first Changes of the Ova of the Mammifera, in consequence of Impregnation, and on the Mode of Origin of the Chorion. By T. W. JONES, Esq. 2
5. Researches in Embryology. By MARTIN BARRY, M.D., F.R.S.E. . . ib.
6. Embryogénie Comparée. Cours sur le Developpement de l'Homme et des Animaux, fait au Museum d'Histoire Naturelle de Paris. Par M. COSTE, et publié sous les yeux du Professeur par les soins de MM. Z. GERBE et V. MEUNIER ib.
Comparative Embryogeny. A Course of Lectures on the Development of Man and Animals, delivered at the Museum of Natural History of Paris, by M. COSTE, and published by MM. Z. GERBE and V. MEUNIER, under the Inspection of the Professor.
7. De Organis quæ Respirationi et Nutritioni Fœtus Mammalium inserviunt. Prolusio Academica, quam scripsit DAN. FREDERICUS ESCHRICHT, M.D. . ib.
On the Organs which are subservient to the Respiration and Nutrition of the Fœtus of Mammalia. By DAN. FREDERIC ESCHRICHT, M.D.

ART. II.—Questioni di Medicina Legale, secondo lo spirito delle Leggi Civili e Penali veglianti nei Governi d'Italia. Del Dottore GIACOMO BARZELLOTTI . 35
Questions in Medical Jurisprudence, based on the Civil and Criminal Laws of Italy. By Dr. G. BARZELLOTTI.

ART. III.—1. Statistical Report of the Sickness, Mortality, and Invaliding among the Troops in the West Indies . . . . . 63
2. Statistical Report of the Sickness, Mortality, and Invaliding among the Troops in the United Kingdom, the Mediterranean, and British America . ib.

ART. IV.—1. Ueber Kuhpocken an Kühen. Von E. HERING . . 76
On Cowpock in the Cow. By E. HERING.
2. Praktische Abhandlung über die Wiedererzeugung der Schutzpockenlymphe durch Uebertragung derselben auf Rinder und andere impffähige Hausthiere. Von Dr. CARL GOTTLOB PRINZ. . . . . . ib.
A Practical Treatise on the Regeneration of the Protecting-pock (Vaccine) Lymph, by transferring it to Heifers and other domestic animals susceptible of inoculation. By Dr. CHARLES GOTTLOB PRINZ.
3. Die Menschen-und Kuhpocken in ihrer Identität und Ruckbildung ersterer zur Vaccine, &c. Von Dr. BASIL THIELE . . . . ib.
On the Identity of Smallpox and Cowpox, and the Reproduction of the latter from the former, &c. By Dr. BASIL THIELE.
4. Report of the Section appointed to enquire into the present State of Vaccination, read at the Anniversary Meeting of the Provincial Medical and Surgical Association, held at Liverpool, July 25, 1839 . . . ib.

§

PAGE

ART. V.—1. The Nervous System of the Human Body; as explained in a Series of Papers read before the Royal Society of London. With an Appendix of Cases and Consultations on Nervous Diseases. By Sir CHARLES BELL . 96

2. Narrative of the Discoveries of Sir C. Bell in the Nervous System. By A. SHAW ib.

3. Documents and Dates of Modern Discoveries in the Nervous System . ib.

ART. VI.—1. Des Etablissemens consacrés aux Aliénés en France, et des Moyens de les améliorer. Par E. ESQUIROL, &c. &c. . . . 144

Of Establishments for the Insane in France, and the Means of Improving them. By E. ESQUIROL, &c. &c.

2. Maisons d'Aliénés. (Dictionnaire des Sciences Médicales.) Par E. ESQUIROL. ib.

Lunatic Asylums. (Dictionary of Medical Sciences.) By E. ESQUIROL.

3. Essai sur les Distributions et le Mode d'Organisation, d'après un Systême Physiologique, d'un Hôpital Aliénés pour quatre à cinq cent Malades; précédé de l'Exposé succinct de la Pratique Médicale des Aliénés de l'Hospice de l'Antiquaille de Lyon. Par R. PASQUIER, D.M., &c. &c. . . ib.

Essay on the Distribution and Organization of a Lunatic Hospital for four or five hundred Patients, according to a Physiological System; preceded by a succinct Account of the Treatment of the Insane in the Hospital of Antiquaille of Lyons. By R. PASQUIER, M.D., &c.

4. On the Constitution of Asylums, and the Mode of their Management. By Sir W. C. ELLIS, M.D. . . . . . . ib.

5. Total Abolition of Personal Restraint in the Treatment of the Insane. A Lecture on the Management of Lunatic Asylums, and the Treatment of the Insane, delivered at the Mechanics' Institution, Lincoln, on the 21st June, 1838 ib.

6. State of the Lincoln Lunatic Asylum. (Fifteenth Report.) . . ib.

7. Sixth Annual Report of the Trustees of the State Lunatic Hospital, Worcester (Massachusetts) . . . . . . ib.

8. Nineteenth Annual Report of the Directors of the Dundee Royal Asylum for Lunatics . . . . . . . ib.

9. Medical Report to the Managers of the Lunatic Asylum of Aberdeen, for the year ending 30th April, 1839 . . . . . ib.

10. Report of the Directors of the Montrose Lunatic Asylum, Infirmary, and Dispensary, for the year ending 1st June, 1839 . . . . ib.

11. State of an Institution near York, called the Retreat, for Persons afflicted with Disorders of the Mind . . . . . . ib.

12. Regulations of the Crichton Institution for Lunatics, Dumfries . . ib.

13. Statistics of the Bloomingdale Asylum for the Insane. By J. MACDONALD, M.D. 145

14. Twenty-ninth Annual Report of the State of the General Lunatic Asylum, near Nottingham, delivered by the Vice-President and Visiting Governors, at their Anniversary Meeting, on Tuesday, the 8th of October, 1839 . ib.

15. Fiftieth and Fifty-first Report of the Visiting Justices of the County Lunatic Asylum at Hanwell, and Report of the Resident Physician, October 31, 1839 ib.

ART. VII.—1. On the Origin of the Moral Qualities and Intellectual Faculties of Man, &c. By FRANÇOIS JOSEPH GALL, M.D. . . . 190

2. Phrenology, or the Doctrine of the Mental Phenomena. By J. G. SPURZHEIM ib.

3. Traité de Phrénologie Humaine et Comparée. Par J. VIMONT, M.D., &c. . ib.

4. Cours de Phrénologie. Par F. J. V. BROUSSAIS . . . ib.

Lectures on Phrenology. By F. J. V. BROUSSAIS, M.D.

5. A System of Phrenology. By GEORGE COMBE . . . ib.

6. Elements of Phrenology. By GEORGE COMBE . . . ib.

7. The Constitution of Man considered in Relation to External Objects. By GEORGE COMBE . . . . . . . ib.

8. On the Functions of the Cerebellum. By Drs. GALL, VIMONT, and BROUSSAIS ib.

9. The Phrenological Journal. Nos. I.—LXI. . . . . ib.

10. Selections from the Phrenological Journal. Edited by R. COX . . ib.

11. Statistics of Phrenology. By HEWITT C. WATSON. . . . ib.

12. An Introduction to Phrenology. By ROBERT MACNISH, LL.D. . . ib.

13. The Principles of Phrenology. By SIDNEY SMITH . . . ib.

14. A Treatise on Insanity and other Disorders affecting the Mind . . ib.

15. Phrenology Vindicated and Anti-Phrenology Unmasked. By C. CALDWELL, M.D. ib.

16. Observations on Mental Derangement. By ANDREW COMBE, M.D. . 191

17. Compendio di Anatomia Fisiologico-Comparata, &c. Del Dr. FILIPPO UCCELLI ib.

Compendium of Physiological and Comparative Anatomy, &c. By Dr. P. UCCELLI.

PAGE
18. Lettera al Signor Defendente Sacchi, sul Merito e Valore della Craniologia . 191
Letter to Signor Defendente Sacchi, on the Merits and Truth of Craniology.
19. Memorie risguardanti la Dottrina Frenologica ed altre Scienze che con essa hauno stretto rapporto. Da Luigi Ferrarese, d.m. . . . ib.
Memoirs on the Phrenological Doctrines and other relative sciences. By Luigi Ferrarese.
20. Caractères Phrénologiques et Physiognomoniques des Contemporains les plus célèbres selon les systêmes de Gall, Spurzheim, &c. Par Theodore Poupin ib.
The Phrenological and Physiological Character of the most celebrated Men of the present day, according to the system of Gall, &c. By Theodore Poupin.
21. Two Treatises on Physiology and Phrenology. By P. M. Roget, m.d. . ib.
22. On the Physiology of the Brain as the Organ of the Mind. By C. Cowan, m.d. ib.
23. Medical Notes and Reflections. By Henry Holland, m.d. . . ib.

Art. VIII.—The Transactions of the Provincial Medical and Surgical Association 216

Art. IX.—Rendiconto Clinico per gli Anni Accademici, 1835-7. Di C. G. Sachero 223

Clinical Report for the Academic Years 1835-7. By C. G. Sachero.

Art. X.—1. Cure of Club-foot, Bent-knee, Wry-neck, Spinal and other Deformities. By Gustav Krauss, m.d. . . . . 227
2. Beiträge zur subcutanen Orthopädie, oder über die Heilung angeborner oder erworbener Contracturen der Glieder mittelst Durchschneidung der verkürzten Sehnen und Muskeln unter der Haut. By Dr. Dieffenbach . ib.
Contributions to the Subject of Subcutaneous Orthopædia, or the Cure of congenital or acquired Contractions of Joints, by division of the shortened tendons and muscles under the skin. By Dr. Dieffenbach.

Art. XI.—Cases of Hydrocephalus or Water in the Head By J. F. Barnard . 230

Art. XII.—Report of the Malignant Fever, called the Pali Plague. By Dr. Ranken 234

### Part Second.—Bibliographical Notices.

Art. I.—Practical Observations, showing that Mercury is the Sole Cause of what are termed Secondary Symptoms. By P. J. Murphy, m.d. . . 237

Art. II.—Nuovo Trattato completo della Gotta in genere. Opera di G. Ciani . 238
A new and complete Treatise on Gout, and its Varieties. By Joseph Ciani.

Art. III—The Modern Treatment of Syphilitic Diseases, both primary and secondary. By Langston Parker, m.r.c.s. . . . . 239

Art. IV.—Tea ; its Effects, Medicinal and Moral. By G. G. Sigmond, m.d. . 241

Art. V.—Outlines of Military Surgery. By Sir George Ballingall, m.d. . 243

Art. VI.—Practical Observations on the Causes and Treatment of Curvatures of the Spine. By Samuel Hare . . . . . 244

Art. VII.—Elements of Physiology. By J. Müller, m.d. . . . ib.

### Part Third.—Selections from the British and Foreign Journals.

### I. THE FOREIGN JOURNALS.

#### ANATOMY AND PHYSIOLOGY.

Signor Novati on the Inoculation of Animals with the Blood of Cholera Patients 245

Petrifaction of the Tissues . . . . . . 246

#### PATHOLOGY, PRACTICAL MEDICINE, AND THERAPEUTICS.

Prof. Brera's Case of Lithotripsy performed by water of Royal Fountain of Recoaro ib.
Dr. Du Bois's Case of Remarkable Tendency to Hemorrhage in a family . 247
Dr. Rasi's Singular Case of Absorption of Milk . . . . 248
M. Maisonneuve's Case, in which a Salivary Calculus was Developed in the Submaxillary Duct, or Canal of Wharton . . . . ib.

PAGE
Dr. Briquet on the Influence of Mercurial Preparations in Smallpox and Cowpox 249
Case of Ileus cured by Injection of Air . . . . . 250
M. Monod on Disease of the Cæcum; Ileus and Gastrotomy . . ib.
Simon on the Structure and Site of Condylomata . . . . 251
Dr. Longhi's Case of Neuralgia, after Amputation, cured by Acupuncture . ib.
M. Cristin on the Use of Acetate of Morphia in Arthritis and Nervous Affections 252
M. Picard's Cases of Epilepsy treated with Belladonna . . . ib.

SURGERY.

M. Bonnet on the Treatment of Varix of the Inferior Extremities . . ib.
Dr Sani on Neuralgia of the Inferior Dental Nerve, cured by Section, &c. . 258
Dr. Kochanowski's Case of Idiopathic Mydriasis . . . ib.
M. Amussat on a new Method of making an artificial Anus in the Lumbar Region 259
M. Jobert's Treatment of External Cancer by Ligature of the Vessels, &c. . 260
Dr. Mercier on a new Method of Detecting Changes in the Prostate Gland . ib.

MIDWIFERY.

Dr. Longhi's Case of Metritis with Epilepsy . . . . 261
M. Cazenave's Case of Impacted Head . . . . . 262
Dr. Hatin's Case of Occlusion of the Neck of the Uterus during Parturition . 263

MEDICAL JURISPRUDENCE AND POLICE, TOXICOLOGY.

Dr. Heyfelder's Case of a Person found Hanged . . . . 264

M. Orfila's Signs of Death from Hanging . . . . 266

Dr. Marchal's Case of Poisoning by Rotten Eggs . . . . 267

Dr. Steinbeck's Case of Suspected Poisoning . . . . 268

M. Sandras on the Poisonous Effects of Copaiba . . . . ib.

### II. THE AMERICAN AND COLONIAL JOURNALS.

### PATHOLOGY, PRACTICAL MEDICINE, AND THERAPEUTICS.

Dr. Reese on the Use of Arsenic in Chorea . . . . 269

SURGERY.

Dr. Heard's Report of Cases of Un-united Fracture . . . . ib.

### III. THE BRITISH JOURNALS.

### ANATOMY AND PHYSIOLOGY.

J. Paget on the Influence of Madder in the Bones of growing Animals . 272

PATHOLOGY, PRACTICAL MEDICINE, AND THERAPEUTICS.

Dr. Thomson on Alkaline Indigestion . . . . . 273
Dr. Corrigan's Observations on the Treatment of Acute Rheumatism by Opium . 274
Dr. Hall's Outlines of a Case of Acute Anasarca and Convulsion . . 276
Dr. Malcolmson's Remarks on Cases of Liver Abscess presenting externally . ib.

SURGERY.

Dr. Brunker's Case of Popliteal Aneurism, successfully treated by pressure . 277

MEDICAL STATISTICS.

Statistics of Poisoning in England . . . . . 278

### Part Fourth.—Medical Intelligence.

Medical Reform . . . . . . . 281
Reform of the London College of Surgeons . . . . 287
North of England Medical Association . . . . . 290
M. Magendie's Experiments on Endosmosis of the Egg . . . ib.
Mr. Wharton Jones's Researches in Embryology . . . . 291
Revaccinations in the Danish Army in 1837-8 . . . . 292
OBITUARY—Drs. Hamilton, Kreysig, Beck, Rutter, Sweatman, M'Divit, and Sir A. Halliday, m.d. . . . . . ib.
Books received for Review. . . . . . 297

